# 105 Inappropriate ICD Shocks in a Patient with Dilated Cardiomyopathy and Broca's Aphasia

**DOI:** 10.1155/2019/8302591

**Published:** 2019-02-14

**Authors:** Christian Georgi, Michael Neuß, Viviane Möller, Martin Seifert, Christian Butter

**Affiliations:** Heart Center Brandenburg-Department of Cardiology and Medical School Brandenburg Theodor Fontane, Bernau bei Berlin, Germany

## Abstract

With a growing number of ICD recipients, device complications are seen more frequently in the clinical setting and outpatient departments. Among the most severe are ICD infections and inappropriate therapies caused by oversensing of atrial tachycardias or lead fracture. We report on a 76-year-old female patient with dilative cardiomyopathy and Broca's aphasia after stroke, who experienced 105 consecutive inappropriate ICD shocks due to cluster missensing of her fractured ICD lead. The diagnosis was complicated and delayed by patient's aphasia emphasizing the need for intensified remote monitoring along with regular in-person visits, especially in people with intellectual or communication disabilities.

## 1. Introduction

The implantable cardioverter-defibrillator (ICD) has been proven to be an effective therapy in the primary and secondary prevention of sudden cardiac death [1].

Yet inappropriate ICD therapies, mainly due to oversensing or SVTs, remain a great problem in device therapy. A lately published meta-analysis reports of inappropriate therapies in around 10% of patients with ICDs within the first year after implantation [2]. It is well known that ICD shocks, both appropriate and inappropriate, have a negative impact on morbidity and mortality [3, 4].

Beyond mortality rates, ICD shocks, especially inappropriate therapies, are found to cause a decline in the quality of life and reduced daily activity and increased general anxiety in postshock patients [5].

A reduction of inappropriate therapies therefore should be achieved by intensive treatment of the underlying disease, optimal ICD programming, and close monitoring of patients at high risk.

In this context, remote monitoring (RM) is a promising complement to conventional in-clinic follow-ups with the potential to dramatically reduce inappropriate therapies [6], although structural deficits concerning data overload, functional responsibility, and imprecise workflows need to be improved. In particular, for people in rural areas, in patients with impaired mobility or mental status, RM offers the chance for improved safety and quality of life. So far, remote control has not been fully implemented in the follow-up of ICD patients in Northern America and Europe, though [7].

We report on an aphasic patient with more than one hundred inappropriate shocks within a few hours that could have been prevented by more frequent expert consultations and connecting her to a remote monitoring program.

## 2. Case Report

A 76-year-old female patient was admitted to our emergency department early in the morning with suspected acute coronary syndrome. The patient had suffered from a major stroke causing Broca's aphasia three months prior to this admission and was referred to us from a nearby neurorehabilitation clinic. Initial ECG showed no signs of acute ischemia, but troponin I levels were about 1000-fold elevated. History taking was complicated by patient's aphasia, but she did not appear to be in acute pain at the time of admission.

**Figure 1:**
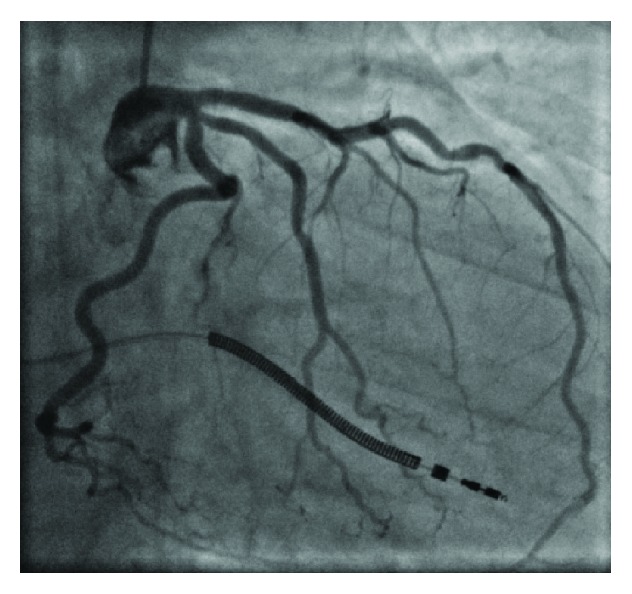
Angiogram of the patient coronaries showing no culprit lesion or significant stenosis.

With a history of heart failure and an implanted single-chamber ICD, the patient was brought to the catheter lab to undergo coronary angiogram, where no culprit lesion could be detected (Figure 1).

In a phone consultation with the rehab clinic's doctor in charge, he described how the patient had multiple episodes of acute chest and back pain with “electrical twitches” for the course of several hours during the past night. Pain medication was administered and the pain interpreted as musculoskeletal but no other diagnostic or therapeutic steps were taken. Eventually, in the morning, a troponin test was done and found positive, so the patient was referred.

**Figure 2:**
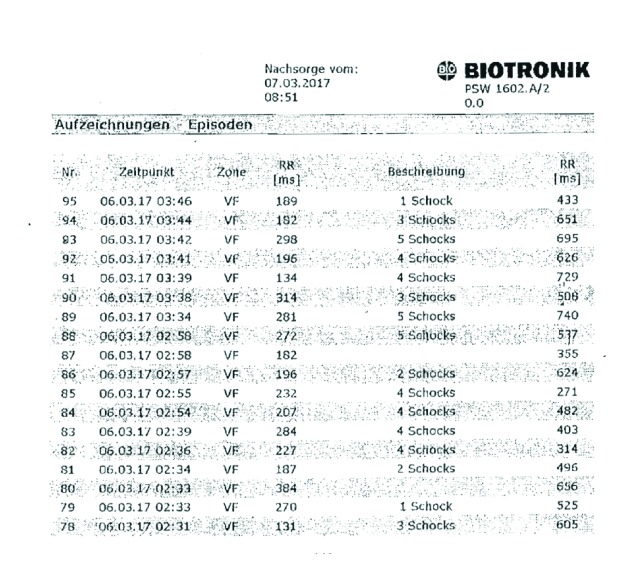
Last page of the ICD memory showing 54 shocks in 1:15 h.

**Figure 3:**
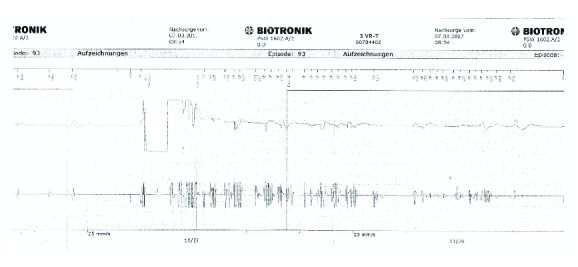
Inappropriate shock. Typical high-frequency signals (cluster) in the PS channel indicating a lead insulation problem.

Subsequently, we performed an ICD interrogation, which revealed an EOS (end of service) status and multiple inappropriate ICD therapies in the time between 00:07 AM and 03:46 AM until the battery of the Biotronik ICD was depleted and the device eventually stopped antitachycardia therapy. In summary, the patient suffered 105 consecutive inappropriate ICD shocks within 219 minutes (Figure 2), to our knowledge, the highest shock incidence in such a short period of time. The shocks were caused by cluster missensing on her right ventricular lead (Figure 3), presumably resulting from an insulation defect near the header. Further episodes of oversensing due to clusters could be seen over the preceding five months, occasionally followed by antitachycardia pacing but no shock therapy.

The ICD was implanted in 2008 and exchanged for EOL (end of life) in 2015. The last ambulatory interrogation was in September 2016, just before the first episodes of cluster missensing occurred. The next appointment was scheduled for March 2017 but postponed due to the prolonged hospital stay after apoplexy. The technical analysis of the explanted ICD did not show any technical abnormalities; the chest X-ray revealed no sign of lead fracture.

After discussing the case with patient's family, the defective lead was disconnected, and at the request of the patient and her family, a new ICD and lead were implanted and the patient enrolled in our remote monitoring program.

## 3. Discussion

Since the first transvenous ICD was implanted in 1980 [8], there is a steadily growing number of ICD implantations and patients with long-lasting devices [9]. In the year 2023, there is an estimated 1.4 million pacemaker and ICD implantations worldwide [10]. Simultaneously, the lead- and generator-associated complications increased over the last years. Lead-associated complications include thrombosis and infection as well as fracture and insulation problems with the risk of inappropriate therapies. From large clinical trials, we know that about 5-20% of patients with ICDs receive inappropriate therapy, mainly due to the missensing of supraventricular arrhythmias, oversensing of external noise, or lead fracture/insulation defects [4]. The psychological impact of inappropriate ICD shocks was investigated in several studies. Among the most frequent side effects are anxiety disorders, posttraumatic stress disorders, panic attacks, depression, nightmare, and insomnia [11].

**Figure 4:**
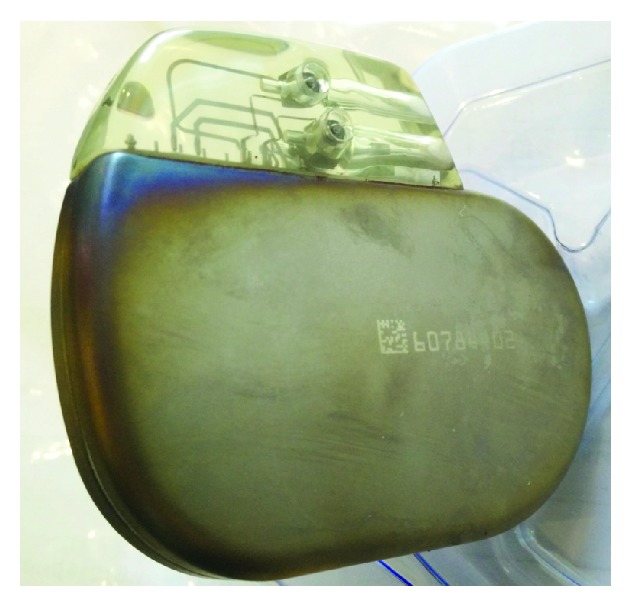
Scorched battery of the Biotronik ICD (Iforia 3 VR and Linox SD lead) due to an assumed lead insulation defect near the header.

This case demonstrates possible pitfalls of ICD supply in elderly or handicapped people. The inability of the patient to communicate properly and missing awareness of the staff led to the dreadful course of events. The suspected short circuit between the lead and the scorched battery (Figure 4) might have reduced the current delivered to the whole body and weakened the pain; still the delay in therapy was unnecessary and avoidable. Immediate ECG monitoring would have helped to discover the cause for shock delivery and could have led to shock suppression by simply applying a magnet.

Retrospectively, the earlier access to remote monitoring could have prevented the massive amount of shocks, since the first asymptomatic cluster episodes could be detected already some months before the incident described. A significant reduction of the “first-incidence-to-action time” through remote monitoring has been described by many authors [12–14]. The TRUST study demonstrated a median delay of 1 day from occurrence of the event to physician evaluation, compared to 1 month with conventional follow-up [15]. They also confirmed a cost benefit from an economical perspective. Still the need for additional manpower to analyze the huge amount of data, legal issues, and lack of standardization remain open problems [16].

## 4. Conclusion

Regular in-person visits with cardiologists remain the foundation of appropriate ICD follow-up. Remote monitoring programs, though, are a very useful tool to supplement conventional follow-up and should be established whenever suitable. The transmission of technical data to experts is known to be very effective to detect early malfunctions of implantable devices as ICDs and pacemakers. This clearly helps to avoid inappropriate therapies, detects lead and battery problems, and discovers atrial fibrillation or other arrhythmias.

An increasing number of patients with ICDs, pacemakers, and CRTs require sufficient manpower and technical resources to guarantee high-quality monitoring. Moreover, further training programs for outpatient departments and GPs should be installed since there is a significant lack of knowledge concerning ICD function and troubleshooting.
